# Quality of data collection in a large HIV observational clinic database in sub-Saharan Africa: implications for clinical research and audit of care

**DOI:** 10.1186/1758-2652-14-3

**Published:** 2011-01-20

**Authors:** Agnes N Kiragga, Barbara Castelnuovo, Petra Schaefer, Timothy Muwonge, Philippa J Easterbrook

**Affiliations:** 1 Research department, Infectious Diseases Institute, P.O. Box 22418 Kampala, Uganda; 2 SURE program, Management Services for Health Uganda Office, P. O. Box 71419, Kampala, Uganda

## Abstract

**Background:**

Observational HIV clinic databases are now widely used to answer key questions related to HIV care and treatment, but there has been no systematic evaluation of their quality of data. Our objective was to evaluate the completeness and accuracy of recording of key data HIV items in a large routine observational HIV clinic database.

**Methods:**

We looked at the number and rate of opportunistic infections (OIs) per 100 person years at risk in the 24 months following antiretroviral therapy (ART) initiation in 559 patients who initiated ART in 2004-2005 and enrolled into a research cohort. We compared this with data in a routine clinic database for the same 559 patients, and a further 1233 patients who initiated ART in the same period. The Research Cohort database was considered as the reference "gold standard" for the assessment of data accuracy. A crude percentage of underreporting of OIs in the clinic database was calculated based on the difference between the OI rates reported in both databases.

We reviewed 100 clinic patient medical records to assess the accuracy of recording of key data items of OIs, ART toxicities and ART regimen changes.

**Results:**

The overall incidence rate per 100 person years at risk for the initial OI in the 559 patients in the research cohort and clinic databases was 24.1 (95% CI: 20.5-28.2) and 13.2 (95% CI: 10.8-16.2) respectively, and 10.4 (95% CI: 9.1-11.9) for the 1233 clinic patients. This represents a 1.8- and 2.3-fold higher rate of events in the research cohort database compared with the same 599 patients and 1233 patients in the routine clinic database, or a 45.1% and 56.8% rate of underreporting, respectively. The combined error rate of missing and incorrect items from the medical records' review was 67% for OIs, 52% for ART-related toxicities, and 83% and 58% for ART discontinuation and modification, respectively.

**Conclusions:**

There is a high rate of underreporting of OIs in a routine HIV clinic database. This has important implications for the use and interpretation of routine observational databases for research and audit, and highlights the need for regular data validation of these databases.

## Background

Prospective research cohorts of HIV-infected persons have made a major contribution to an understanding of the transmission, natural history and pathogenesis of HIV infection [1-3], in addition to generating important information on the response to and long-term outcomes with antiretroviral therapy (ART). Distinctive features of these research cohorts are their voluntary enrolment of selected eligible patients, prospective follow up and standardized data collection at regular defined time points. Their principal disadvantages are that they are costly to establish and maintain, tend to study selected populations, and may be poorly representative of the demography and outcomes of the majority of patients currently receiving ART.

As a result, over the past decade, there has been a major shift towards the use of local or regional observational HIV clinic databases to answer key questions related to HIV care and treatment. These are usually based on unselected patients in care at a single clinic or across multiple clinics, and use data collected during the routine delivery of HIV care [4-6]. Their key advantages are that they are generally large and representative, since they are based on all patients in care, and involve minimal additional resources as a result of using routinely collected data. Their principal limitations are missing, incomplete or inaccurate data due to either visit schedules varying according to patient need, or to failures in either data collection or data entry. Although some of these databases have quality assurance and auditing programmes [4,7], there has been no systematic evaluation of the quality of data in these now widely used HIV observational databases in different clinical settings.

The Infectious Diseases Institute (IDI) in Kampala, Uganda, is a centre of excellence for HIV clinical care in the country, and maintains a large observational clinic database and a nested research cohort database on HIV-1-infected patients registered for care. Our objective was to assess the quality of data collection in a large HIV observational routine clinic database by evaluating the completeness and accuracy of recording several key data items, including opportunistic infections (OIs), ART drug toxicities, and reasons for ART regimen change or discontinuation.

## Methods

### Clinic database

From 1 January 2000 to 31 December 2007, 19,577 HIV-infected patients had registered for care at IDI, of whom 13,099 remained in active follow up and 6421 had started ART. We focused on the subgroup of 1792 clinic patients who initiated ART over the period, 1 April 2004 to 30 April 2005. Of these, 559 patients were also consecutively enrolled into a nested research cohort, and 1233 patients received care through the routine clinic alone.

The electronic capture of all information onto the clinic database began in November 2005. Key data items recorded by clinicians at monthly follow-up visits included: OIs, ART regimen, ART toxicities, and reasons for treatment modification. Full blood count and CD4+ T cell count tests were performed every six months; HIV-RNA measurements were not performed routinely, but could be requested when treatment failure was suspected.

At IDI, clinic antiretroviral treatment is prescribed according to World Health Organization (WHO) 2006 and Uganda Ministry of Health guidelines [8]. The first-line ART for adults and adolescents is stavudine or zidovudine, plus lamivudine, and nevirapine or efavirenz. On average, 350 patients were seen daily by 10 to 15 physicians. In addition to recording details of the clinic visit in the patients' medical record, the selected data items were also recorded by the physician on a clinic monitoring summary sheet, from which they were entered by a data administrator into an electronic database.

### Research cohort

From 1 April 2004 to 30 April 2005, in the initial phase of the ART rollout in Uganda, 1792 patients registered at IDI were eligible for ART initiation. Of these, 559 were consecutively enrolled into a prospective research cohort. Reasons for not enrolling in the research cohort included patient refusal or various exclusion criteria e.g., residing more than 8 km from IDI, and previous ART exposure.

Research study visits took place every three months, in addition to the routine monthly clinic visits. The data items collected were similar to those at the routine clinic visits, but standardized data collection forms were used, and there was additional information collected on sexual behaviour, adherence and quality of life. On average, about 15 research cohort patients were seen daily by two trained study physicians, and data were entered into a separate research cohort database by a dedicated research clerical team. Full details of the research cohort study procedures have been described previously [9].

All research participants had data from their monthly routine clinic visits recorded on the routine clinic database, but data collected at research visits on patients co-enrolled in both the clinic and the research cohorts were not entered onto the clinic database. All CD4 cell counts for both the research and routine clinic patients were carried out by a single laboratory: Makerere University-Johns Hopkins Uganda collaboration laboratory, which is accredited by the College of American Pathologists.

### Outcomes and statistical analyses

We compared the baseline demographic and clinical characteristics at ART initiation for the 559 patients enrolled into the Research Cohort study with the 1233 patients who were initiated on ART between 1 April 2004 and 31 April 2005, but were not enrolled into the research cohort. Categorical variables were compared using Chi-square test, while the Mann-Whitney test was used for the continuous variables [10]. We also examined the baseline characteristics in all 6421 patients who had ever initiated ART at IDI to assess how representative our study populations were of all patients on ART.

We calculated incidence rates of OIs per 100 person years at risk (100 PYAR), where duration of follow up was based on time from ART initiation to date of development of OI or death, or date of their last clinic or research cohort study follow-up visit closest to 24 months (731 calendar days), if the patient was not under "active" follow up [10]. For the patients who were still under follow up as of December 31 2007, the date of their last routine clinic visit closest to 24 months of follow up or at the 24-month visit in the research cohort was used. The overall incidence rate for all initial OIs, and for each OI, was calculated.

We undertook two different comparisons of the rate of OIs in the first 24 months following ART initiation with the reference "gold standard" research cohort database to determine the completeness and accuracy of key data items recorded in the routine clinic database. First, we compared the OI rate for the 559 patients in the routine clinic database with their records in the research cohort database (Comparison A). The second analysis (Comparison B) compared the 1233 patients in the routine clinic database with the 559 patients in the research cohort database in order to assess the level of underreporting in a larger number of patients in the clinic database who were not part of a research study.

For each analysis, we compared the overall incidence rates of the initial OI, and then the rate for the nine most common OIs individually. These included tuberculosis, severe bacterial pneumonia, herpes zoster, oral and oesophageal candidiasis, cerebral toxoplasmosis, Cryptococcus meningitis, genital herpes, Kaposi's sarcoma and an "other OI" category (*Pneumocystis jirovecii *pneumonia, CMV retinitis, lymphoma, HIV-related anaemia, septicaemia, chronic diarrhoea, intracerebral mass, and pulmonary aspergillosis).

A crude percentage of underreporting of OIs in the clinic database was calculated using two approaches: the absolute number of OI events (only for the same 559 patients in the clinic and research databases); and the incidence rate of OI events per 100 PYAR in the first 24 months following ART initiation. We based this on the difference between either the number of OI events or incidence rates reported by the research cohort database and the routine clinic database, divided by the gold standard reference research cohort rate, multiplied by 100, as reported by Ricci *et al *[11]. The second approach was necessary as a direct comparison using absolute number of events in the research cohort and routine clinic databases was not possible for the 1233 patients, given that the two groups of patients were not the same.

We used a further strategy to determine the completeness and accuracy of data collected in the routine clinic database by comparing the documentation (missing or incomplete) on the summary sheets of 100 randomly selected patients on ART in the clinic database versus that contained in their medical records for: the nine main OIs; ART toxicities (peripheral neuropathy, anaemia, neutropaenia, rash, efavirenz-related side effects, headache, nausea, diarrhoea, nail discolouration, lipodystrophy, lactic acidosis, and abnormal liver function); and reasons for change or discontinuation of ART regimen (toxicity, intolerance, treatment failure, and co-morbidity, e.g., tuberculosis). We calculated a total error rate based on the combined number of missing and incorrect events that were found in the clinic database after cross validation with the information found in the patients' medical records.

## Results

Table 1 summarizes the baseline demographic, clinical and laboratory characteristics at ART initiation for: the 559 patients enrolled in the research cohort who initiated ART between April 2004 and April 2005; the 1233 patients in the routine clinic database who also initiated ART between April 2004 and April 2005, and who were not enrolled into the research cohort; and the 6421 patients in the routine clinic database who had ever initiated ART in the clinic up to 31 December 2007. Research cohort study patients had similar proportions of women (64% vs. 62% and 64%, respectively, p = 0.417), were slightly older (38 vs. 37 and 36 years, p < 0.060), and had more advanced WHO stage disease (stage 3-4 (89% vs. 79% and 71%, p < 0.0001). Overall, the 6421 patients in the routine clinic database had a higher CD4 count (121 cells/mm^3^) compared with the subset of patients of 1233 and 559 patients who initiated ART over the same period: they had a similar median CD4 count of 95 cells/mm^3 ^and 98 cells/mm^3^, respectively.

**Table 1:** Baseline characteristics at ART initiation for patients in the research cohort and routine clinic databases

**Characteristic**	**Research** **cohort database of** **patients initiated on** **ART between April** **2004 and April 2005** **(n = 559)**	**Routine clinic** **database of patients** **initiated on ART** **between April 2004** **and April 2005** **(n = 1233)**	**Routine clinic** **database of all** **patients initiated on** **ART between January 2000 and** ** December 2007** **(n = 6421)**	**P value **^**a**^
Female, n (%)	386 (64%)	765 (62%)	4112 (64%)	0.417
Age in years, median (IQR)	38 (33, 44)	37 (32, 43)	36 (31, 42)	0.064
WHO stage III & IV, n (%)	496 (89%)	975 (79%)	4581 (71%)	<0.0001
CD4 cells/mm^3^, median (IQR)	98 (21, 163)	95 (25, 168)	121 (147, 187)	0.232
ART regimen, n (%)				
d4T+3TC+nevirapine	414 (74%)	727 (59%)	4224 (66%)	<0.0001
ZDV+3TC+efavirenz	145 (26%)	506 (41%)	2117 (34%)	

ART = Antiretroviral therapy, WHO World Health Organization, d4T = stavudine, AZT = Zidovudine, 3TC = lamuvudine, IQR = Interquartile range

**a**.Based on comparison between 559 patients enrolled into the research cohort and the 1233 initiated on ART over the same time period in the clinic database, but not enrolled into the research cohort. P value derived from Chi-square test across proportions for the categorical variables (female, WHO stage and ART regimen) and Mann-Whitney test for continuous variables (age and CD4+ cell count).

Table 2 shows the rates of OIs for the nine most frequent first OIs in the 24 months after ART initiation among the same 559 patients in the research cohort and routine clinic databases, and the 1233 patients in the routine clinic database who had initiated ART between April 2004 and April 2005.The overall incidence rate 100 PYAR (resulting 95% confidence intervals) of the initial OI in the 24 months after ART initiation in the 559 patients in the research cohort and clinic databases was 24.1 (20.5-28.2) and 13.2 (10.8-16.2), respectively, and 10.4 (9.1-11.9) for the 1233 patients in the clinic database. This represents a 1.8- and 2.3-fold higher rate of events in the research cohort database compared with the same 599 patients and the 1233 patients in the routine clinic database, or a 45.2% and 56.8% rate of underreporting, respectively, compared with the research cohort.

**Table 2:** Number and incidence rates of opportunistic infections in the initial 24 months after ART initiation, and percent underreporting of OI events in routine clinic versus research cohort databases for: (Comparison A) 559 patients in research cohort and same 559 patients in routine clinic databases; and (Comparison B) 559 patients in research cohort database versus 1233 patients in routine clinic database

	**Research cohort database** **(n = 559)**		**Routine clinic database** **(n = 559)**		**Routine clinic database** **(n = 1233)**		**% underreporting of OI** **events in 559 patients in** **routine clinic vs.** **research cohort database** **(Comparison A)**		**% underreporting of OI** **events in 1233 patients in** **routine clinic vs.** **research cohort database** **(Comparison B)**
Type of OI	No. of OI events	Incidence rate (95% CI (per 100 PYAR)	No. of OI events	Incidence rate (95% CI (per 100 PYAR)	No. of OI events	Incidence rate (95% CI (per 100 PYAR)	Based on absolute number of OI events	Based on OI rates	Based on OI rates
Overall (initial OI only)	154^a^	24.1 (20.5- 28.2)	91^a^	13.2 (10.8 - 16.2)	206^a^	10.4 (9.1 - 11.9)	40.9	45.2	56.8
Oral candidiasis	63	8.4 (6.5 - 10.7)	26	3.7 (2.5 - 5.4)	54	2.6 (2.0 - 3.3)	58.7	55.9	69.0
Severe bacterial pneumonia	44	5.5 (4.1 - 7.6)	8	1.0 (0.5 - 2.1)	4	0.2 (0.1 - 0.5)	81.8	81.8	96.4
Tuberculosis	33	4.1 (2.9 - 5.8)	27	3.6 (2.5 - 5.3)	73	3.6 (2.8 - 4.5)	18.2	12.2	12.2
Herpes zoster	29	3.6 (2.5 - 5.2)	5	0.6 (0.3 - 1.3)	9	0.4 (0.2 - 0.8)	82.7	83.3	88.8
Cryptococcus meningitis	7	0.8 (0.4 - 1.8)	7	0.9 (0.4 - 1.9)	16	0.7 (0.4 - 1.2)	0	+12.5 ^b^	12.5
Genital herpes	5	0.6 (0.2 - 1.4)	15	2.0 (1.2 - 3.3)	12	0.5 (0.3 - 1.0)	+200 ^b^	+233^b^	16.7
Kaposi's sarcoma	5	0.6 (0.2 - 1.4)	5	0.6 (0.3 - 1.6)	31	1.5 (1.1 - 2.2)	0	+16.7 ^b^	+150 ^b^
Oesophageal candidiasis	5	0.6 (0.2 - 1.4)	0	0.0 (0.0 - 0.0)	4	0.2 (0.1 - 0.5)	100	100	66.7
Cerebral toxoplasmosis	2	0.2 (0.1 - 0.9)	0	0.0 (0.0 - 0.0)	4	0.2 (0.1 - 0.5)	100	100	0
Other OIs^c^	8	0.9 (0.5 - 1.9)	3	0.4 (0.1 - 1.2)	11	0.5 (0.3 - 0.9)	62.5	55.5	44.4

**a **represents the total number of initial OI events only. In the research cohort database of 559 patients, there were 154 initial OIs, but a total of 204 events, as 116 had one OI, 28 had two OI events, and 10 had three or more OIs. In routine clinic database of 559 patients, there were 91 initial OIs, but at total of 100 events, as 87 had one OI, seven had two OI events, and one patient had three OIs. In routine clinic database of 1233 patients, there were 206 initial OIs, but a total of 224 events, as 188 had one OI, 16 had two OI events, and two patients had three OIs.)

**b **OIs where there were more events reported in the clinic versus research database, representing underreporting in the research database.

**c **Other OIs includes *Pneumocystis jirovecii *pneumonia, CMV retinitis, lymphoma, HIV-related anaemia, septicaemia, chronic diarrhoea, intracerebral mass, and pulmonary aspergillosis.

Of note, the underreporting percentage for the overall number of initial OIs calculated using absolute number of OI events and incidence rates was similar, (40.9% vs. 45.2%, respectively). Similarly, the underreporting percentage for all the individual OIs calculated using absolute number of OIs and incidence rates was similar and was within 5% of each other for most of the individual OIs, except tuberculosis and genital herpes, for which there were a higher number of events recorded in the routine clinic database. We therefore considered calculation of underreporting based on incidence rates as a valid approach for the further comparison with the 1233 patients in the routine clinic database. Furthermore, the 559 research cohort participants and the 1233 patients in the routine clinic database (n = 1233) were comparable in gender, age, baseline CD4 count and WHO stage, although slightly more of the clinic patients were initiated on efavirenz-based regimens.

High percentages of underreporting of OIs in the 559 and 1233 patients in the routine clinic database were recorded for severe bacteria pneumonia (81.8% and 96.4%, respectively), herpes zoster (83.3% and 88.8%), oral candidiasis (55.9% and 69.0%) and oesophageal candidiasis (100% and 66.7%). There were low (<20%) rates of underreporting or even better reporting in the routine clinic compared with the research cohort database for the more serious life-threatening OIs of tuberculosis (12.2% and 12.2%), *Cryptococcus *meningitis (+12.5% and 12.5%), and Kaposi's sarcoma (+16.7% and +150%). Although the same number of patients was identified with *Cryptococcus *meningitis and Kaposi's sarcoma among the 559 patients in both databases, the incidence rate in the clinic database was slightly higher because of the smaller number of person years in the routine clinic database (769.2 years versus 847.5 years in the research cohort database). There was also a higher incidence and 233% better reporting of genital herpes in the routine clinic than in the research cohort database.

### Data audit

In the audit of quality of data on OIs, toxicities and treatment discontinuation or modification in the routine clinic database based on a medical records review of 100 randomly selected patients on ART, the baseline characteristics at ART initiation in the 100 patients were similar to those in the overall clinic population of 1233 patients: 67% female, median age (IQR) of 37 (31, 43) years and median CD4 (IQR) of 83 (39, 160) cells/mm^3^.

Overall, the number (%) of missing and incorrect entries in the clinic database was 124 (55%) and 27 (12%) of 127 OIs identified; 220 (49%) and 15 (3%) of 453 toxicities, and 18 (51%) and 11 (32%) of the 86 cited reasons for ART discontinuation and modification. This gives a total error rate (comprising missing and incorrect items) of 67% for OIs, 52% for ART-related toxicities, and 83% and 58% for ART discontinuation and modification, respectively. Nineteen of the 559 patients in the research cohort were included in the data audit exercise, and we identified five OI events in the audit. All these events had already been correctly captured in the research cohort database, validating the quality of data capture of OIs in the research cohort database.

## Discussion

In a large HIV observational clinic database of patients receiving ART in Uganda, we found an overall high level of underreporting for all OIs combined (45.1 and 56.8%), based on a comparison with a nested research cohort that had more intensive and standardized data collection procedures. The level of underreporting was particularly high (>80%) for severe bacterial pneumonia, herpes zoster and oesophageal candidiasis, using several methods and approaches for calculating underreporting The level of underreporting was significantly less for the more serious life-threatening OIs, such as tuberculosis (18.2%) and Cryptococcus meningitis (0%), which we attributed to patients being more likely to be on ongoing treatment and prophylaxis at their clinic visit, which would be noted by the supervising physician.

There are several potential reasons for this underreporting. We examined whether this was due to a true difference in the incidence of OIs as a result of more advanced disease in the research cohort participants at ART initiation in comparison with the 1233 patients in the routine clinic database. However, both groups had a similar CD4 count of 95 cells/mm^3 ^at ART initiation. The underreporting was also not explained by temporal differences in OI rates as patients in the research cohort database initiated ART over the same time period as the 1233 patients in the routine clinic, and we also directly compared the OI events recorded in the same 559 patients in the clinic and research cohort databases.

In the further validation exercise involving an audit of 100 randomly selected medical records, we found an error rate of 67% for OIs, 52% for ART related toxicities, and 83% and 58% for ART discontinuation and modification, respectively. From this data audit exercise, we also determined that underreporting of OIs on the database was mainly due to the lack of documentation of the key data items on the summary sheet by the healthcare worker in the setting of a busy clinic, rather than a failure of or incorrect data entry from the summary sheet onto the database. These observations are not unique to HIV clinical observational databases, and poor documentation of co-morbidities in databases that collect information during the routine delivery of care to patients has been well described in different clinic settings, including those that are better resourced and staffed [12-14].

## Conclusions

These findings have important implications for the use and interpretation of data derived from routine HIV observational databases for research and audit, and they highlight the need for ongoing regular validation of key data items in these databases. This evaluation is particularly timely and relevant with the expanding use of observational databases to assess the optimal timing of ART initiation, and the establishment of seven regional International Epidemiologic Databases to Evaluate AIDS (IeDEA) networks of HIV clinical databases to address key questions relevant to HIV care and ART management in resource-limited settings [15].

At present, few publications based on observational clinic data report the strategies used to validate key data items, such as OIs, deaths, toxicities and reasons for ART regimen change. The significant rate of loss to follow up from ART programmes, due in part to unreported mortality, highlights the limitations of conclusions based just on those remaining under follow up [16-19]. The availability in our setting of a nested research cohort employing more intensive and standardized data collection approaches within a larger clinic observational database presented us with a unique opportunity to assess the quality of data collection in the clinic database. However, in most other clinics, the quality of data collection can only be verified through detailed and laborious review of medical notes, which are often poorly organized, missing or illegible.

We have instituted several measures to improve the quality of data collection in the clinic database. First, over the past year, we have undertaken a comprehensive retrospective audit of all OIs, ART toxicities and reasons for ART regimen change, based on the medical records of all 6500 patients on ART and under active follow up, with both retrospective and real-time tracking of patients lost to follow up for clinical outcomes and death [20].

Second, we have introduced weekly electronic downloads of laboratory data, and the use of prescription data on, for example, anti-TB medication, or fluconazole as a means to flag unreported OIs, such as TB, oesophageal candidiasis and Cryptococcus disease.

Third, a comprehensive user-friendly clinic database reference manual has been developed to support induction and periodic training updates of all new clinic staff in proper data collection procedures, which highlights the key data items and codes for abstraction onto the summary sheet. The provision of a summary sheet of key clinically useful data, such as serial CD4 counts and clinical events for each patient to facilitate patient care, provides an important incentive to the healthcare worker to maintain good data collection practices.

Finally, since October 2009, we have introduced a daily real-time prospective monitoring of data capture of 21 key variables at each patient visit by an on-site quality assurance and control team that reviews the medical records of all the patients seen daily at the IDI clinic [21]. Since introduction of the programme in October 2009, there has been a reduction in the percentage of missing and incorrect entries. We would encourage other HIV care programmes to institute similar simple measures to improve the quality of their patient data.

## Competing interests

The authors declare that they have no competing interests.

## Authors' contributions

ANK participated in the design of the study and performed the statistical analysis. BC, PS and TM participated in its design and coordination, and provided comments on the manuscript. PJE conceived the study, participated in its design and coordination, and drafted the manuscript with ANK. All authors have read and approved the final manuscript.

## References

[B1] Kaslow RA Ostrow DG Detels R Phair JP Polk BF Rinaldo CR Jr The Multicentre AIDS Cohort Study: rationale, organization and selected characteristics of the participants American Journal of Epidemiology 1987 14 310 318 3300281 10.1093/aje/126.2.310

[B2] Barkan SE Melnick SL Preston-Martin S Weber K Kalish LA Miotti P Young M Greenblatt R Sacks H Feldman J The Women's Interagency HIV Study Epidemiology 1998 14 117 125 10.1097/00001648-199803000-00004 9504278

[B3] The UK Collaborative HIV Cohort Steering Committee The creation of a large UK-based multicentre cohort of HIV-infected individuals: The UK Collaborative HIV Cohort (UK CHIC) Study HIV Med 2004 14 115 124 10.1111/j.1468-1293.2004.00197.x 15012652

[B4] Zhou J Kumarasamy N Ditangco R Kamarulzaman A Lee CK Li PC Paton NI Phanuphak P Pujari S Vibhagool A Wong WW Zhang F Chuah J Frost KR Cooper DA Law MG The TREAT Asia HIV Observational Database. Baseline and Retrospective Data TREAT Asia HIV Observational Database J Aquir Immune Defic Syndr 2005 14 174 179 10.1097/01.qai.0000145351.96815.d5 PMC3070962 15671802

[B5] Dabis F Balestre E Braitstein P Miotti P Brinkhof WG Schneider M Schechter M Laurent C Boulle A Kabugo C Capkun G Seyler C McIntyre J Sprinz E Bangsberg D Van der Borght S Egger M The Antiretroviral Therapy in Lower Income Countries (ART-LINC) Study Group. Cohort Profile: Antiretroviral Therapy in Lower Income Countries (ART-LINC): international collaboration of treatment cohorts Int J Epidemiology 2005 14 979 986 10.1093/ije/dyi164 16157617

[B6] Phillips A Grabar S Tassie JM Costagliola D Lundgren J Egger M for the EUROSIDA, the French Hospital Database on HIV and the Swiss HIV Cohort Study Groups Use of observational databases to evaluate effectiveness of antiretroviral therapy for HIV infection: comparison of cohort studies with randomized trials AIDS 1999 14 2075 2082 10.1097/00002030-199910220-00010 10546860

[B7] Sabin CA Lampe FC Chaloner C Madge SJ Lipman MC Youle M Phillips AN Johnson MA An audit of antiretroviral treatment use in HIV-infected patients in a London clinic: the limitations of observational databases when auditing antiretroviral treatment use HIV Medicine 2003 14 87 93 10.1046/j.1468-1293.2003.00141.x 12702128

[B8] National antiretroviral treatment and care guidelines for adults and children 2003 1 Kampala, Uganda: Ministry of Health, Republic of Uganda

[B9] Kamya MR Mayanja-Kizza H Kambugu A Bakeera-Kitaka S Semitala F Mwebaze-Songa P Castelnuovo B Schaefer P Spacek LA Gasasira AF Katabira E Colebunders R Quinn TC Ronald A Thomas DL Kekitiinwa A Predictors of long term viral failure among Uganda children and adults treated with antiretroviral therapy J Aquir Immune Defic Syndr 2007 14 187 193 10.1097/QAI.0b013e31814278c0 17693883

[B10] Rothman KJ Modern Epidemiology 1986 Boston: Little, Brown 164 170

[B11] Ricci M Goldman AP R de Leval M Cohen GA Devaney F Carthey J Pitfalls of adverse event reporting in paediatric cardiac intensive care Arch Dis Child 2004 14 856 859 10.1136/adc.2003.040154 15321866 PMC1763185

[B12] Preen DB Holman CD Lawrence DM Baynham NJ Semmens JB Hospital chart review provided more accurate comorbidity information than data from a general practitioner survey or an administrative database Journal of Clinical Epidemiology 2004 14 1295 1304 10.1016/j.jclinepi.2004.03.016 15617956

[B13] Humphries HK Rankin MJ Carere GR Buller EC Kiely MF Spinelli JJ Co-morbidity data in outcomes research. Are clinical data derived from administrative databases a reliable alternative to chart review? Journal of Clinical Epidemiology 2000 14 343 349 10.1016/S0895-4356(99)00188-2 10785564

[B14] Norris C Ghali W Knudtson M Naylor D Saunders D Dealing with missing data in observational health care outcome analyses Journal of Clinical Epidemiology 2000 14 377 383 10.1016/S0895-4356(99)00181-X 10785568

[B15] About IeDEA 2010 http://www.iedea-hiv.org/index.cfm

[B16] Brinkhof MWG Dabis F Myer L Bangsberg DR Boulle A Nash D Schechter M Laurent C Keiser O May M Sprinz E Egger M Anglaret X for the ART-LINC of IeDEA collaboration Early loss of HIV-infected patients on potent antiretroviral therapy programmes in lower-income countries Bull World Health Organ 2008 14 7 559 567 10.2471/BLT.07.044248 18670668 PMC2647487

[B17] Geng EH Emenyonu N Bwana MB Glidden DV Martin JN Sampling-based approach to determining outcomes of patients lost to follow-up in antiretroviral therapy scale-up programs in Africa JAMA 2008 14 5 506 507 10.1001/jama.300.5.506 18677022 PMC3711416

[B18] An MW Frangakis CE Musick BS Yiannoutsos CT The need for double-sampling designs in survival studies: an application to monitor PEPFAR Biometrics 2009 14 1 301 306 10.1111/j.1541-0420.2008.01043.x 18479488 PMC4137787

[B19] Brinkhof MW Pujades-Rodriguez M Egger M Mortality of patients lost to follow-up in antiretroviral treatment programmes in resource-limited settings: systematic review and meta-analysis PLoS One 2009 14 6 e5790 10.1371/journal.pone.0005790 19495419 PMC2686174

[B20] Muwanga A Easterbrook PJ Schaefer P Wandera M Okello D Castelnuovo B Kamya M Kambugu A Losses to Follow-up in a Large ART Program in Uganda. [abstract #840] 15th Conference on Retroviruses and Opportunistic Infections (CROI), Boston, MA 2008

[B21] Birabwa E Matovu E Achieng M Kambugu A Easterbrook PJ The role of prospective continuous data quality improvement (CQI) strategies in improving data quality for an HIV clinic database in Uganda XVII International AIDS Conference, Vienna, Austria 2010 Abstract No. MOPDE101

